# Transcriptome Analysis of Insulin Signaling-Associated Transcription Factors in *C. elegans* Reveal Their Genome-Wide Target Genes Specificity and Complexity

**DOI:** 10.3390/ijms222212462

**Published:** 2021-11-18

**Authors:** Neha Kaushik, Soumya Rastogi, Sonia Verma, Deepak Pandey, Ashutosh Halder, Arnab Mukhopadhyay, Neeraj Kumar

**Affiliations:** 1 Department of Reproductive Biology, All India Institute of Medical Sciences, Ansari Nagar, New Delhi 110029, India; neha1993kaushik@gmail.com (N.K.); soumya.rastogi05@gmail.com (S.R.); deepakpandey@aiims.edu (D.P.); ashutoshhalder@gmail.com (A.H.); 2 Division of Neuroscience and Ageing Biology, CSIR-Central Drug Research Institute, Lucknow 226031, India; sonia.verma1@cdri.res.in; 3 Molecular Aging Laboratory, National Institute of Immunology, Aruna Asaf Ali Marg, New Delhi 110067, India; arnab@nii.ac.in

**Keywords:** FOXO/DAF-16, NRF-2/SKN-1, HSF1/HSF-1, insulin-IGF-1 signaling, *daf-2*, *C. elegans*, transcriptomics, RNAi

## Abstract

Insulin/IGF-1-like signaling (IIS) plays a crucial, conserved role in development, growth, reproduction, stress tolerance, and longevity. In *Caenorhabditis elegans,* the enhanced longevity under reduced insulin signaling (rIIS) is primarily regulated by the transcription factors (TFs) DAF-16/FOXO, SKN-1/Nrf-1, and HSF1/HSF-1. The specific and coordinated regulation of gene expression by these TFs under rIIS has not been comprehensively elucidated. Here, using RNA-sequencing analysis, we report a systematic study of the complexity of TF-dependent target gene interactions during rIIS under analogous genetic and experimental conditions. We found that DAF-16 regulates only a fraction of the *C. elegans* transcriptome but controls a large set of genes under rIIS; SKN-1 and HSF-1 show the opposite trend. Both of the latter TFs function as activators and repressors to a similar extent, while DAF-16 is predominantly an activator. For expression of the genes commonly regulated by TFs under rIIS conditions, DAF-16 is the principal determining factor, dominating over the other two TFs, irrespective of whether they activate or repress these genes. The functional annotations and regulatory networks presented in this study provide novel insights into the complexity of the gene regulatory networks downstream of the IIS pathway that controls diverse phenotypes, including longevity.

## 1. Introduction

The evolutionarily conserved insulin/insulin-like growth factor (IGF)-1 signaling (IIS) pathway is among the best-characterized genetic network that regulates aging and a host of other biological functions in various organisms ranging from simple invertebrates to mammals [1]. In *C. elegans*, the reduced IIS condition (rIIS) exhibited by *daf-2* receptor mutants controls these processes at the transcriptional level primarily by its three TFs, namely, DAF-16/FOXO, SKN-1/Nrf (NF-E2-related factor), and HSF-1 (heat shock transcription factor 1) [2,3,4]. Under optimal conditions, IIS sequesters the two prominent transcription factors (TFs), DAF-16 and SKN-1, within the cytoplasm in their inactive forms through a series of well-organized phosphorylation events. These events do, however, cease in reduced IIS receptor signaling (rIIS), leading to the reversal of cytoplasmic sequestration and translocation of the TFs into the nucleus, where they engage in transcriptional regulation [2,3]. However, HSF-activation appears to be a tightly controlled multistep process that includes oligomerization, posttranslational modifications, nuclear localization, and acquisition of DNA binding activity [5,6]. In *C. elegans*, many of these HSF-1 activation processes are negatively regulated by insulin signaling by the formation of a DDL-1-containing HSF-1 inhibitory complex (DHIC) that seems to reduce the pool of HSF-1 susceptible to heat stress stimulation. Reduced IIS activity promotes DDL-1 phosphorylation and disrupts DHIC formation, and consequently, increases HSF-1 activity under heat-stressed and unstressed conditions [7].

The leading output of rIIS, DAF-16, belongs to the FoxO family of Forkhead transcription factors, which are known to regulate differentiation, metabolism, proliferation, and survival [8]. In *C. elegans*, DAF-16 is also responsible for a dramatic increase in life span, stress tolerance, higher fat stores, and has a proclivity to arrest at an alternate developmental stage called dauer on rIIS [9,10,11]. Various DAF-16/FOXO targets in the rIIS condition were identified by multiple approaches such as bioinformatics predictions [12], microarrays [13,14,15,16], serial analysis of gene expression (SAGE) [17], protein mass spectrometry [18], DamID [19], RNA-Seq, and ChIP-Seq [20,21], although precise regulation and function of numerous other genes remain to be explored.

SKN-1, best known as a regulator of antioxidant and xenobiotic defense, is an ortholog of the Nrf (NF-E2-related factor)/CNC family of transcription regulators [3,22]. During the earliest embryonic stages, it initiates the development of the feeding and digestive system, but in the post-embryonic period, its role shifts towards controlling normal lifespan and stress resistance [3,23,24]. SKN-1 promotes lifespan by maintaining protein homeostasis through the regulation of the proteasome machinery [25,26,27]. Constitutive nuclear overexpression of SKN-1 also extends lifespan in a DAF-16/FOXO-independent manner [3]. Under rIIS, it extends longevity parallel to DAF-16 but in a genetically distinct scenario from the dauer pathway [28]. Genome-wide binding sites of SKN-1 were revealed by the modENCODE consortium using ChIP-Seq in *C. elegans* larval stages [29]. SKN-1 expression profiling under the rIIS condition in *C. elegans* has been reported by a single study using microarray, which is based on predefined transcripts/genes [28,30]. Therefore, the full spectrum of genome-wide targets needs to be revealed by using highly specific and sensitive probe-independent alternative technologies.

HSF-1, another important TF, acting downstream of IIS, is implicated in processes, including stress resistance, development, metabolism, and longevity [4,7,31,32,33,34,35]. Shortening and extension of lifespan were reported to be dependent on genetic ablation and overexpression of *hsf-1*, respectively [7,31,36,37]. The knockdown of *hsf-1* also suppressed the enhanced longevity phenotype of the *daf-2(e1370)* strain [4]. Genome-wide binding sites of HSF-1 in *C. elegans* were reported in L2 larval stages by ChIP-Seq [38]. The role of HSF-1 in the regulation of gene expression was reported through RNA-Seq in wild-type adults [39], L2 larvae [38], and overexpressed *hsf-1* young adult *C. elegans* animals [40]; however, HSF-1 genome-wide transcriptional targets under rIIS condition still need to be explored. 

Here, we elucidate the genome-wide transcriptional complexity of the principal TFs under rIIS using RNA-Seq in a comparable genetic and experimental setting. Our study provides a comprehensive framework to understand the transcriptional interplay by IIS-associated TFs under analogous conditions.

## 2. Results

### 2.1. Characteristics Concurrently Modulated by TFs under rIIS

**Figure 1:**
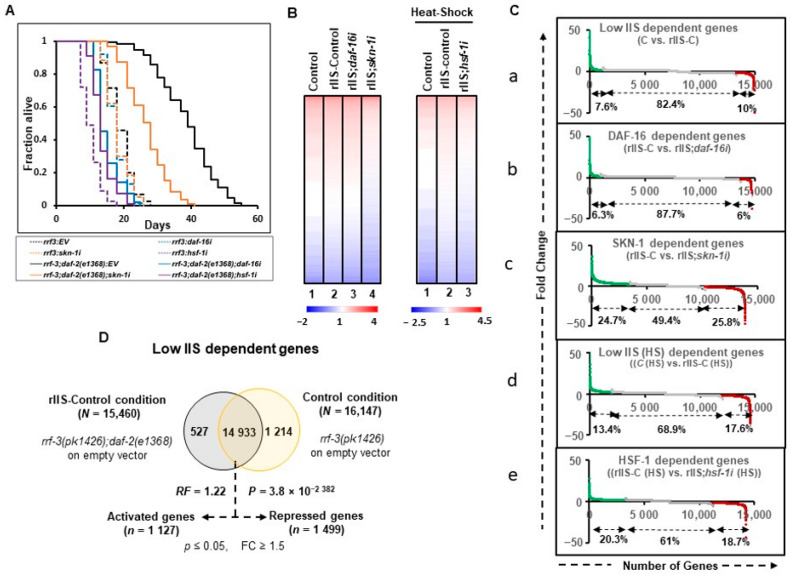
Global transcriptional outputs of transcription factors affecting class 1 rIIS receptor mutant longevity. (**A**) Life-spans of mutants *rrf-3(pk1426)* and *rrf-3(pk1426);daf-2(e1368)* on empty vector (EV), *daf-16, skn-1,* and *hsf-1* RNAi. Strains of *rrf-3(−)* and *rrf-3(−);daf-2(−)* on the empty vector (EV) were marked as “control (C)” and ‘rIIS-Control (rIIS-C)”, respectively. The knockdown of *daf-16* and *hsf-1* genes reverts the lifespans of rIIS mutant (*rrf-3(−);daf-2(−)*) close to control levels, while it partially depends on *skn-1* RNAi. (**B**) DAF-16, SKN-1 and HSF-1dependent genes were identified by feeding *rrf-3(−);daf-2(−)* animals on *daf-16, skn-1* and *hsf-1* RNAi, respectively. Heat-map representation of the total gene expression by RNA-Seq (log_10_ RPKM values) contributing to (left panel) control: lane 1, rIIS: lane 2, DAF-16 dependent genes: lane 3, and SKN-1 dependent genes: lane 4, (right panel) control (HS): lane 1, rIIS (HS) dependent genes: lane 2, and HSF-1 (HS) dependent genes: lane 3. Strong knockdown of TFs by the RNAi represented by the lowered expression values in TFs knockdown conditions compared to rIIS. (**C**) Significant fold change, (**a**) rIIS vs. control at 20 °C, (**b**,**c**) rIIS vs. rIIS mutant on *daf-16, skn-1* RNAi, (**d**) rIIS (HS) vs. control (HS) at 33 °C for 2 h, and (**e**) rIIS (HS) vs. rIIS (HS) on *hsf-1* RNAi. At the genome-wide level, differentially expressed genes due to DAF-16 are less than that of SKN-1 and HSF-1. Activated and repressed genes were shown by green and red colors, respectively. (**D**) Venn diagram shows the overlap among genes that are differentially expressed during low IIS conditions. Genes selected with *p* ≤ 0.05 and fold change ≥1.5. P: Hypergeometric *p*-value, HS: heat-shock, RF: representation factor, FC: fold change.

**Figure A1:**
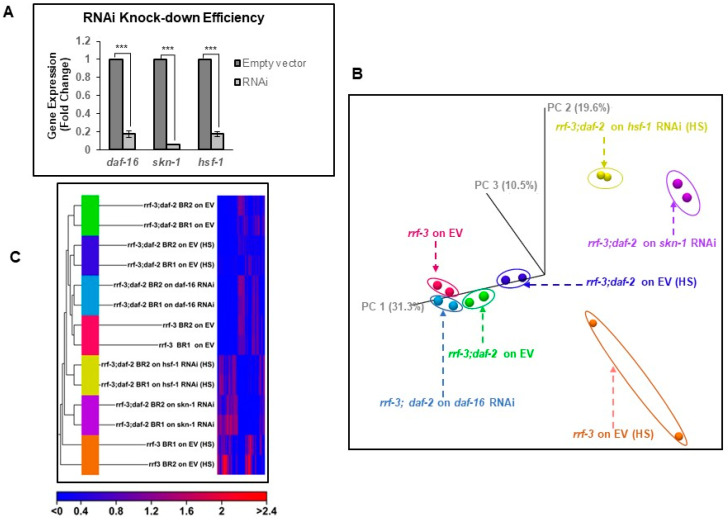
RNAi knockdown efficiency and data quality parameters. (**A**) RNA expression of the *hsf-1* genes was reduced by 80%, while for *skn-1* genes, it was reduced by more than 90%. (**B**) Principal component analysis (PCA) provides insights into the association between biological replicates. (**C**) Unsupervised hierarchical condition tree analysis clustered all the biological replicates under the same branch indicates a high degree of reproducibility with similar gene expression between biological replicates. *** *p* ≤ 0.001.

**Figure A2:**
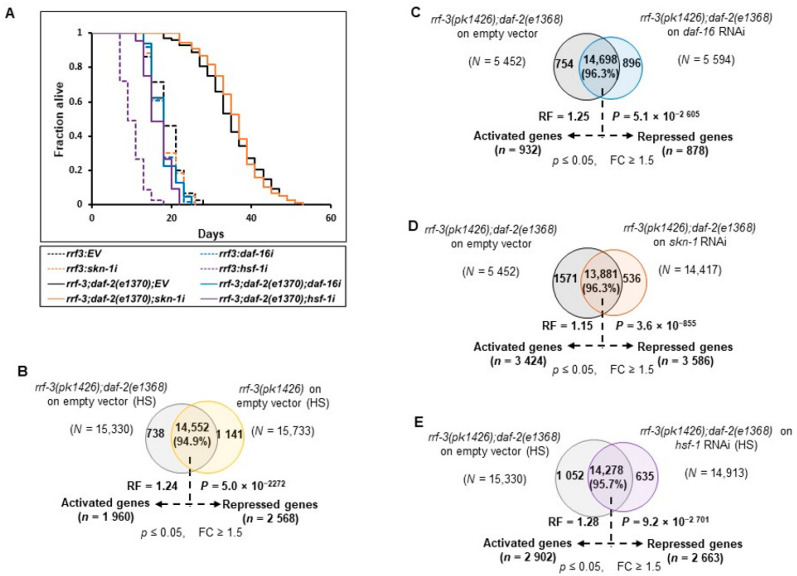
Lifespans of *daf-2* class 2 allele and genome-wide transcriptional output of transcription factors. (**A**) Lifespan of *daf-2* stronger class 2 allele does not depend on all three transcription factors. Life-spans of mutants *rrf-3(pk1426)* and *rrf-3(pk1426);daf-2(e1370)* on empty vector, *daf-16, skn-1,* and *hsf-1* RNAi. Knock-down of *daf-16* and *hsf-1* genes reverts the life-spans of rIIS mutant [*rrf-3(-);daf-2(-)*] near to control levels but *skn-1* knockdown does not suppress the life-span of *rrf-3(pk1426);daf-2 (e1370)* mutants. (**B**–**E**) Venn diagram shows the overlap among genes that are differentially expressed during low IIS (+HS), *daf-16, skn-1,* and *hsf-1* knockdown conditions respectively.

To select comparable genetic and experimental conditions, *C. elegans’* strain, temperature, and developmental stages were considered, which represent the effects of all TFs on the well-discerned longevity phenotype. We examined two widely used *daf-2* alleles with moderate (e1368, class 1) or strong (e1370, class 2) phenotypes [41]. The studies on double mutant *daf-2(e1370)* and *hsf-1(sy441)* were avoided as *hsf-1* mutant has an egg-laying and temperature-sensitive developmental arrest phenotype [33]. The longevity phenotype of the class II allele, *daf-2(e1370),* has been reported to be mainly dependent on DAF-16 and HSF-1 [4,15]. On the other hand, the longevity of the class I allele, *daf-2(e1368),* primarily depends on DAF-16 and SKN-1 [3,41]. We observed that all TFs included in this study regulate the longevity phenotype in e1368 (class1) but not in the e1370 (class 2) allele (Figure 1A and Figure A2A). Therefore, the class I allele *daf-2(e1368)* was used for further revelation of the underlying gene complexity. To knock down the specific TFs efficiently, RNAi hypersensitive double mutant strain *rrf-3(pk1426);daf-2(e1368)* was opted for [42]. TF-specific RNAi clones in each biological replicate were found to be efficient at knocking down their target expression (Figure A1A).

For transcriptomics analysis, samples were collected at L4/Day-1 adult stage after growing the worms at 20 °C on control or TF RNAi, except for *hsf-1* RNAi *(hsf-1i).* Under normal temperature, HSF-1 exists in a monomeric form that, under heat stress, trimerizes and gets transcriptionally activated [43,44]. *C. elegans* can be temperature-stressed by being grown at 32–40 °C [33,45,46,47]. To activate HSF-1, we chose to provide acute heat shock at 33 °C for 2 h, and the animals were harvested immediately. This provides enough time for the induction of the heat-responsive genes without affecting the health of the thermotolerant *daf-2* allele used in the study.

### 2.2. DAF-16 Regulates a Relatively Small Fraction of the Genome-Wide Transcriptional Output

To gain insight into the regulatory functions of the TFs downstream of rIIS, we performed RNA-seq of two independent biological replicates of L4/ young adult (YA) worms after TF-specific RNAi knockdown, starting at the L1 larval stage (Table S1). Principal components analysis (PCA) and unsupervised hierarchical clustering tree analysis that grouped all the biological replicates under the same branch indicate a high degree of reproducibility with similar gene expression between biological replicates (Figure A1B, C). We refer to the *rrf-3(pk1426)* strain on the empty vector (EV) as “Control (C)” and *rrf-3(pk1426);daf-2(e1368)* double mutant on EV as “rIIS-Control (rIIS-C)” conditions. The heat-stressed samples of the same genetic background are referred to as “C (HS)” and “rIIS-C (HS)”.

Visualization of the comprehensive transcriptomic data (log_10_ RPKM values) indicates that only a small fraction of the total genes changes their expression at normal temperatures (20 °C) between control and rIIS conditions, as compared to that of heat-shock conditions (Figure 1B left and right panels: lane-1and lane-2). However, these gene expression patterns appear to revert to the control conditions on *daf-16i* (Figure 1B, left panel: lane 1–3). On *skn-1* and *hsf-1* RNAi, a large proportion of the genes appear to change their expression pattern compared to their respective rIIS conditions (Figure 1B left panel: lane 2 and 4 and right panel: lane 2 and 3). This data indicates that the RNAi of the TFs worked efficiently to bring down the expression levels of most of the genes close to that of the control conditions.

**Figure A3:**
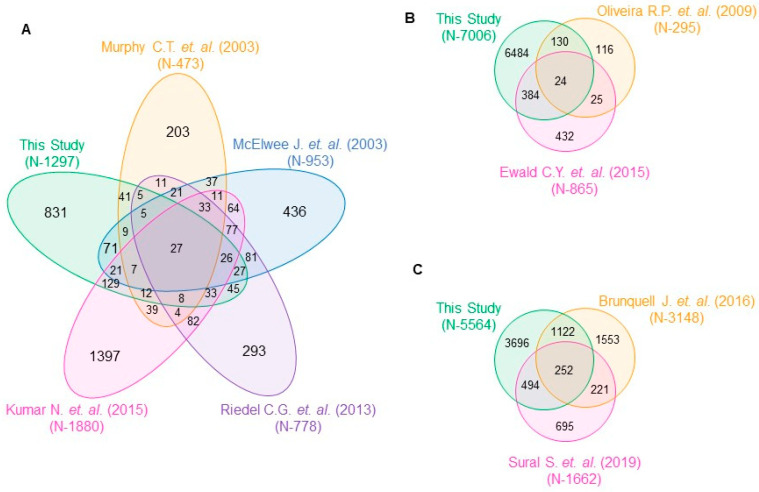
Reduced IIS condition-dependent gene comparison with earlier datasets. Venn diagrams represent the overlap with earlier published datasets. Differentially expressed genes were considered together to compare with earlier published (**A**) DAF-16, (**B**) SKN-1, and (**C**) HSF-1 datasets.

Common genes among different conditions were compared for further analysis and the exclusive genes specific to a single condition were discarded as most of them had low read counts (RPKM < 10). We identified 14,933 (96.6%, RF = 1.22, *P* = 3.8 × 10^−2382^) common genes under rIIS condition (rIIS-C vs. C), 14,552 (94.9%, RF = 1.24, *P* = 5.0 × 10^−2272^) in rIIS (HS) condition [rIIS-C (HS) vs. C (HS)], 14,698 (94.25%, RF = 1.25, *P* = 5.1 × 10^−2605^) in *daf16i* (rIIS-C vs. rIIS;*daf-16i*), 13,881 (96.3%, RF = 1.15, *P* = 3.6 × 10^−855^) in *skn-1i* (rIIS-C vs. rIIS;*skn-1i*) and 14,278 (95.7%, RF = 1.28, *P* = 9.2 × 10^−2701^) in *hsf-1i* [rIIS (HS) vs. rIIS;*hsf-1i* (HS)] condition (Figure 1C, D and Figure A2B–E). Then, differentially expressed genes (*p* ≤ 0.05) with fold change (FC ≥ 1.5) were considered among these common pools of genes (Table S2). In this study, all comparisons were made in such a way that up- and down-regulated genes are termed “activated” and “repressed” genes, respectively. A total of 1127 (7.6%) and 1960 (13.4%) activated, and 1499 (10%) and 2568 (17.6%) repressed, genes were identified specific to rIIS and rIIS (HS) conditions, respectively (Figure 1Ca, D, Cd and Figure A2B). Similarly, DAF-16, SKN-1, and HSF-1 were found to activate 932 (6.3%), 3424 (24.7%), and 2902 (20.3%), and repress 878 (6%), 3586 (25.8%), and 2663 (18.7%) genes, respectively (Figure 1C and Figure A2C–E). This data indicates that DAF-16 regulates a smaller set of the total transcriptional output of the *C. elegans* genome than SKN-1 and HSF-1, hinting towards a more diverse but important role of SKN-1 and HSF-1, compared to DAF-16. These TF-specific genes also showed significant overlap with the previously published literature using stronger class-I allele *daf-2(e1370)* that further strengthens our confidence in our experimental strategy (Figure A3).

### 2.3. DAF-16 Alone or with SKN-1 Regulates the Majority of Genes under rIIS

**Figure 2:**
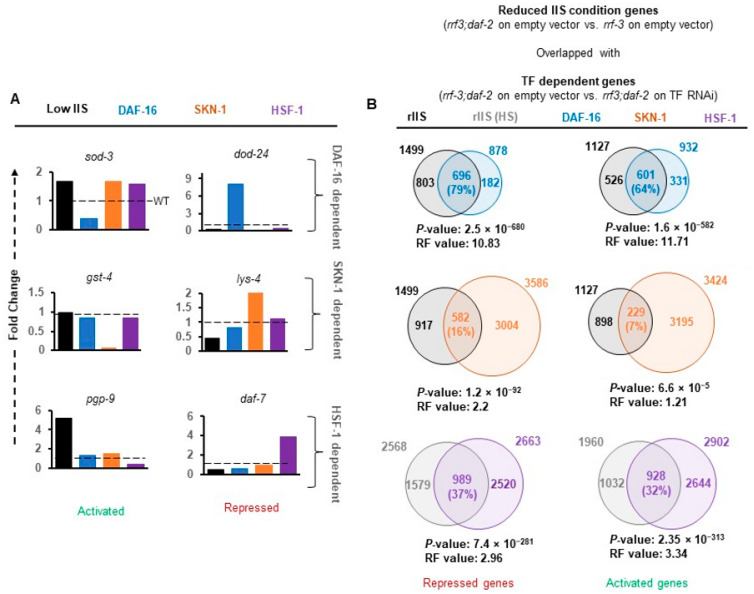
The major proportion of genes under reduced insulin signaling is controlled by DAF-16. (**A**) RNA expression of TFs’ well-established targets. Expression of the genes *sod-3, gst-4,* and *pgp-9* activated by DAF-16, SKN-1, and HSF-1. *lys-4,* and *daf-7* repressed by DAF-16, SKN-1, and HSF-1, respectively, increased without their regulators. (**B**) Venn diagrams represent the number of genes regulated by TFs, specifically under reduced insulin signaling. Major fractions of rIIS-dependent activated (*N* = 601, 64%,) or repressed (*N* = 696, 79%) genes are controlled by DAF-16, followed by HSF-1 (928, 32%) and (989, 37%). Contrarily, a small fraction of SKN-1-regulated genes were either activated (*N* = 229, 7%) or repressed (*N* = 582, 16%). Genes selected with *p* ≤ 0.05 and fold change ≥1.5. P: Hypergeometric *p*-value, HS: heat-shock, RF: representation factor, FC: fold change.

**Figure A4:**
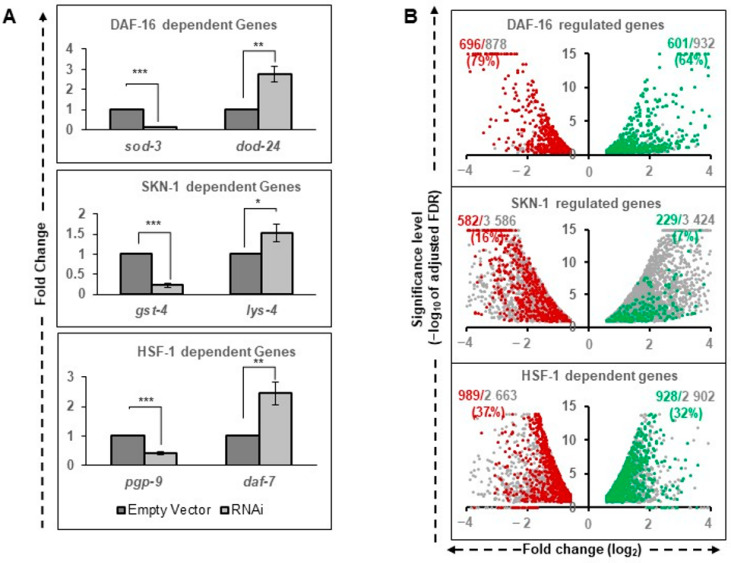
Quantification of rIIS-dependent targets and relative fraction of TF-dependent genes. (**A**) Quantitative real-time PCR of DAF-16 (upper panel), SKN-1 (middle panel), and HSF-1 (lower panel) well-known targets. One target each from the genes activated and repressed by the respective TF has been quantified in the *rrf-3(pk1426);daf-2 (e1368)* double mutants. (**B**) Volcano plots between log_10_ of adjusted FDR significance level and log_2_ fold change of TF-dependent genes (gray color dots). The fraction of these genes specific to the rIIS condition is represented by green and red coloring. Genes regulated by TFs and specific to rIIS condition are shown in percentage. The *p*-values were represented with * ≤0.05, ** ≤0.01 and *** ≤0.001.

To validate our data, we specifically compared the expression levels of well-known targets (identified in the stronger allele of *daf-2*) in our data sets (Figure 2A). Activation of *sod-3* and repression of *dod-24* genes under rIIS condition was found to be entirely dependent on DAF-16 (Figure 2A, upper panel) as reported earlier [15,48]. Similarly, the targets of SKN-1 (*gst-4* and *lys-4*) [3,28]) and HSF-1 (*pgp-9* and *daf-7*) [39,49] were found to be mainly dependent on their respective TFs (Figure 2A, middle and lower panels). Quantitative analysis of these genes further confirmed their dependence on their respective TFs under the rIIS condition (Figure A4A). The above observation suggests that in the moderate *daf-2* allele, TFs regulate transcriptional output, which can be reliably extrapolated to the other rIIS strains.

TF-dependent differential gene expression (rIIS-C vs. rIIS;TF RNAi-referred as TF-regulated genes) reflects the cumulative gene response. To delineate the rIIS-specific contribution, genes changing expression on rIIS condition were compared with TF-regulated genes (Figure 2B). We observed that most DAF-16-regulated genes were either activated (*N* = 601, 64%, RF = 11.71, *P* = 1.6 × 10^−582^) or repressed (*N* = 696, 79%, RF = 10.83, *P* = 2.5 × 10^−680^) under rIIS conditions (Figure 2B and Figure A4B top panels). Contrarily, a small fraction of SKN-1 regulated genes were either activated (*N* = 229, 7%, RF = 1.21, *P* = 6.6 × 10^−5^) or repressed (*N* = 582, 16%, RF = 2.2, *P* = 1.2 × 10^−32^; Figure 2B and Figure A4B middle panels). In the case of HSF-1, nearly 1/3 of the genes were found to be activated (928, 32%, RF = 1.01, *P* = 0.42) and repressed (989, 37%, RF = 1.66, *P* = 2.0 × 10^−13^) under rIIS condition (Figure 2B and Figure A4B lowest panels). Even from the rIIS point of view, 49.4% ((696 + 601)/(1499 + 1127))of rIIS-dependent genes were found to be regulated by DAF-16, 42.3% ((989 + 928)/(2568 + 1960)) by HSF-1, and 31.2% ((582 + 229)/(1499 + 1127)) by SKN-1 (Figure 2B).

**Figure A5:**
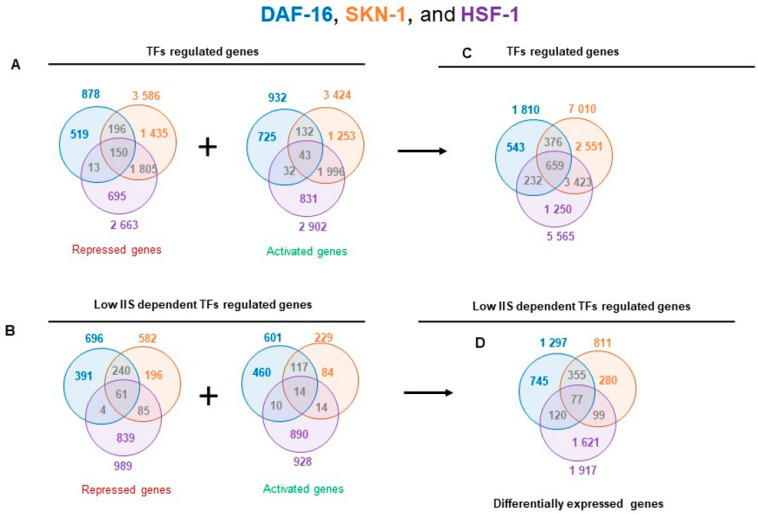
Gene expression profiles of rIIS associated TFs. (**A**) Venn diagram of TF-dependent repressed and activated genes. (**B**) Venn diagram of TF-dependent repressed and activated genes under reduced IIS condition. (**C**) A total of 9034 differentially expressed non-redundant genes regulated by all three TFs. (**D**) A total of 3297 non-redundant differentially expressed genes regulated by all three TFs under rIIS conditions.

**Figure A6:**
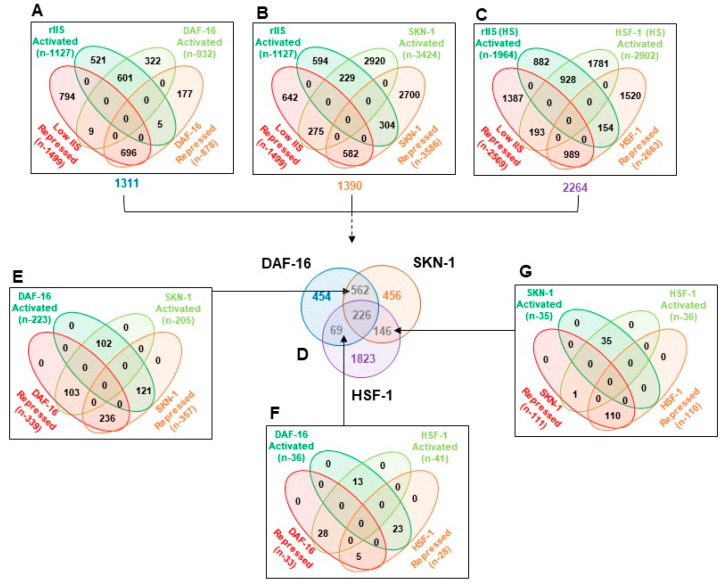
Identification of common genes. (**A**–**C**) Common genes between TFs and rIIS signaling were identified in all possible combinations. DAF-16 regulates a total of 1311, SKN-1-1390, and HSF-1 1831 under reduced IIS conditions. (**D**) Triple Venn diagram shows overlap among TF-regulated genes under rIIS condition. (**E**–**G**) Common activated and repressed genes by any two TFs under rIIS conditions were identified.

To further gain insight into the TF interactions for the regulation of their targets, each cluster of the TF-dependent differentially expressed genes were considered together. We identified activated and repressed genes for each transcription factor knockdown compared to their controls, resulting in a total of 14,385 differentially expressed genes for all transcription factors that correspond to 9034 non-redundant genes (Figure A5A,C). It shows that 4344 (48%) differentially expressed genes were specific to a single TF, 4031 (44.6%) were shared by at least two TFs, and 659 (7.3%) were identified in all TF knockdown conditions (Figure A5C). Under rIIS conditions, these genes fall into 1589 activated and 1816 repressed from the total of 4025 genes (Figure A5B,D), which corresponds to 3297 non-redundant genes (Figure A6D). Further, 2646 (80%) genes were found to be specific for a single TF, 547 (17.4%) by any two TFs, and a very small fraction (77 genes, 2.3%) by all TFs (Figure A6D). This implies that only a small number of genes are regulated jointly by all three TFs, and most of them are regulated independently by a single TF under rIIS condition (Figure A5C,D). Despite a smaller set of genes regulated by SKN-1 under the rIIS condition, it regulates the major set (N-355) of common genes with DAF-16 (Figure A5D), but at the genome-wide level, SKN-1 and HSF-1 were found to regulate the largest set (N-3423) of non-redundant genes (Figure A5C). Together, our data suggest that DAF-16 independently or jointly with SKN-1 regulates the largest fraction of rIIS-dependent genes.

### 2.4. Distinct Activator and Repressor Activities of TFs under rIIS

**Figure 3:**
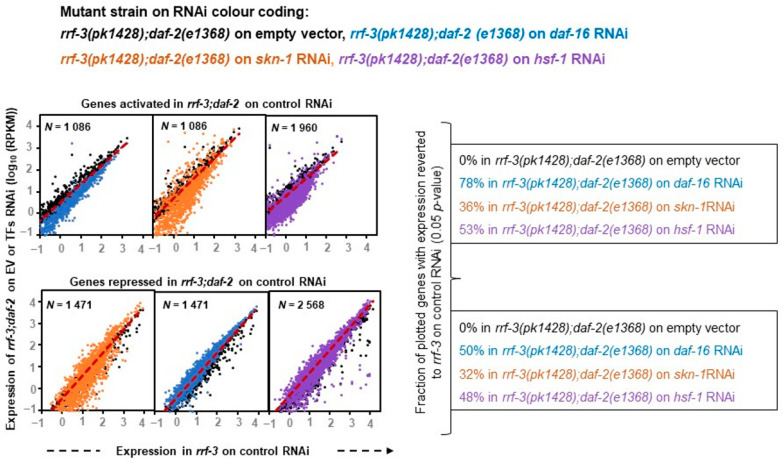
Distinct activator and repressor activities of TFs downstream of insulin signaling. Scatter-plots comparing gene expression (log_10_ RPKM) in control (*rrf-3* mutant on empty vector) with rIIS condition (*rrf-3;daf-2*) double mutant on empty vector) and TF knock-down conditions (*rrf-3;daf-2*) double mutant on *daf-16/skn-1/hsf-1* RNAi). Genes expressed with RPKM ≥ 10 in all comparing conditions either significantly activated (top panel) or repressed (down panel) by *rrf-3(pk1426);daf-2(e1368)* were considered. Colors indicate the strains on RNAi in which the gene expression was analyzed. Gene expression away from the plots’ diagonals represents significantly activated or repressed genes in the rIIS condition compared to control (black dots). The shift of genes near to the plots’ diagonals due to *rrf-3;daf-2* on TFs RNAi represents the fraction of genes with expression reverted to the “control” condition. Mutant *rrf-3;daf-2* on *daf-16* RNAi extensively, *rrf-3;daf-2* on *skn-1* RNAi partially and *rrf-3;daf-2* on *hsf-1* RNAi marginally reverted the rIIS expression near to control levels. As indicated by the large fraction of DAF-16-dependent activation of genes suggests its predominant role as an activator, while SKN-1/HSF-1 shows a similar extent of activation and repression activities.

To comprehend the accurate nature of these transcription factors, we further investigated how effectively TF knockdown can bring the rIIS-dependent differential gene expression close to the control condition. This approach appears to be a more suitable and reliable predictor to understand the exact function of these transcription factors, as it is based on a larger inclusion of datasets that we might have overlooked in our overlap analysis (Figure 2B) owing to stringent and independent comparisons based on fold change. With this approach, we found that RNAi knockdown of DAF-16 extensively reversed the gene expression of rIIS to control levels (78% of activated and 50% of rIIS repressed genes) as reported earlier in the class-2 mutant strain [20] (Figure 3, left panels).

Similarly, SKN-1 and HSF-1 TF data analysis with similar parameters revealed that they significantly reversed almost equal fractions, i.e., 1/3 (36% of activated and 32% of rIIS repressed genes) and 1/2 (53% of activated and 48% of rIIS repressed genes) of differential gene expressions of rIIS, respectively (Figure 3, middle and right panels) indicating that both of them acts as a transcriptional activator and repressor to the same extent, which is in contrast to the above observation. This suggests that DAF-16 controls the majority of the rIIS genes predominantly by acting as a transcriptional activator, as reported earlier [20]. While SKN-1 and HSF-1 both act as transcriptional activators as well as repressors almost to the same extent.

### 2.5. Shared Genes under rIIS Condition Are Predominantly Governed by DAF-16

**Figure 4:**
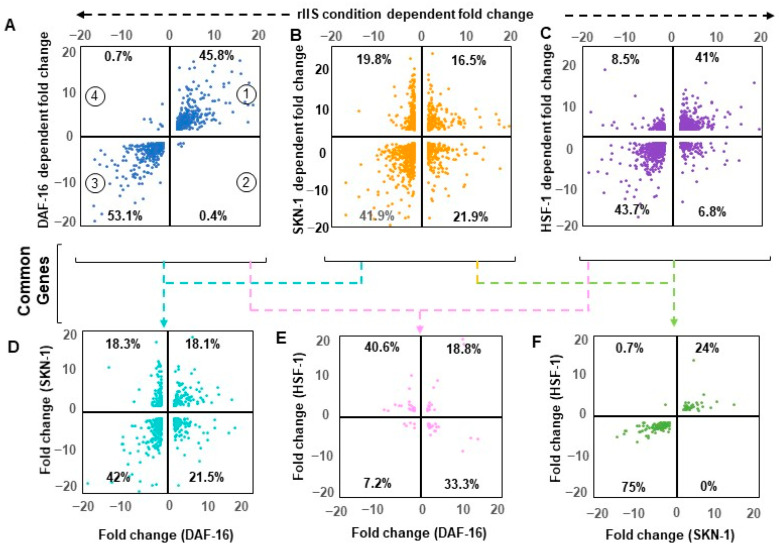
A combinatorial pattern of gene regulation. Quadrant scatter plot depicts all four possible combinations between fold change of activated and repressed genes in two different conditions. Only common genes were considered for analysis. Percentage in each quarter represents the fraction of the total common genes between compared conditions. The upper panel represents a pattern of transcription factor-regulated genes with reduced insulin signaling. Almost all DAF-16 regulated genes partner together with rIIS-dependent genes (**A**). Similarly, most of the HSF-1-regulated genes also aligned with the rIIS condition (**C**), but the SKN-1-regulated genes indicated a mixed pattern (**B**). In the lower panel, common genes between transcription factors under rIIS were considered for analysis (please refer to Figure A6 for detailed analysis). The majority (60%, quarter 1 and 3) of the SKN-1-dependent genes partner together with DAF-16 (**D**), while most of the HSF-1-regulated genes (74%, quarter 2 and 4) behave oppositely to DAF-16 (**E**). SKN-1 and HSF-1 regulate most of their common genes (99%) in the same manner (**F**).

To understand the exact mode of individual TF regulation, all commonly differentiated genes between “rIIS-C” (when all TFs get localized within the nucleus) and knockdown of each TF under rIIS conditions were considered, which in turn, provided four sets of overlapping genes (Figure A6A–C). Quadrant plot of these genes (1311 of DAF-16, 1390 of SKN-1, and 2264 of HSF-1 dependent) revealed that 99% of DAF-16 and 85% of HSF-1-dependent genes are regulated in the same manner as that of the “rIIS-C” condition (Figure 4A,C, quadrant 1 and 3). Surprisingly, genes regulated by these TFs show similar expression patterns under the rIIS condition, despite their slight target overlap (Figure A5D). While in the case of SKN-1, 40% of its genes exhibit a reverse pattern under “rIIS-C” (Figure 4B, quadrant 2 and 4). Such gene expression regulation seems to be dominated by other factors under the rIIS condition.

**Figure 5:**
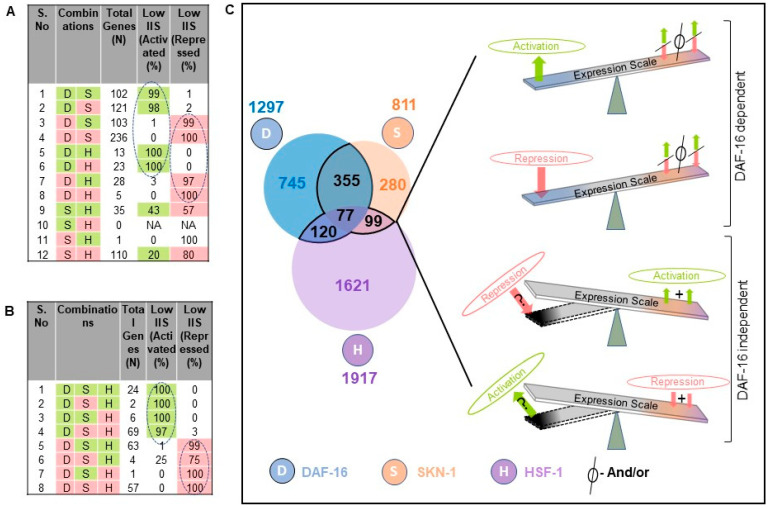
DAF-16 is a key determining factor for the combinatorial regulation of genes. (**A**) Genes are shared by any two TFs. Serial No. 1–8: Differential expression of most genes under rIIS condition (last two columns) are the same as controlled by DAF-16 (first column). Serial No. 9–12: DAF-16-independent genes jointly regulated by SKN-1 and HSF-1. Several genes (57% and 20 %) expressed under the rIIS condition were found to be opposite to the SKN-1 and HSF-1 nature of regulation. (**B**) Genes shared by all three TFs. It shows the dominant nature of DAF-16 under the rIIS condition to determine the direction of expression of the commonly regulated gene by all three TFs. Green and red color boxes indicate activated and repressed genes, respectively. Dotted oval shaped circles represent most of the genes either activated or repressed jointly by any two (**A**) or all three TFs (**B**) under rIIS condition. (**C**) Model for common genes targeted by any two or all three TFs. The relative contribution of TFs is incorporated to provide a nuanced portrayal of the multiple possibilities that cause the expression of gene targets under the rIIS condition. In this view, the effect of TFs on the right side is shown with a seesaw. In the above two panels, overweighted seesaw side indicates DAF-16 dominance irrespective of the contribution made by the other two TFs under the rIIS condition. However, the majority of DAF-16-independent genes are either activated or repressed by SKN-1 or HSF-1 synergistically, but few others behave differently, as indicated by the overweighted dark color side. The result is that wherever DAF-16 is involved, it masks the effect of other TFs.

To penetratingly examine if another TF under this study is dominating in the regulation of genes that behave against the nature of their respective TF under rIIS condition, common targets between any two and all three TFs were further compared (Figure A6D). First, we considered the genes shared by any two TFs under the “rIIS condition” (Figure 4D–F and Figure A6E–G). The distribution of genes regulated by DAF-16 and SKN-1 shows that both these TFs regulate around 60% of their shared genes similarly (Figure 4C, quarter 1 and 3), and the remaining behaves differently (Figure 4C, quarter 2 and 4) while DAF-16 and HSF-1 regulate most of their common targets (74%) inversely (Figure 4E, quarter 2 and 4). However, almost all (99%) of the common genes between SKN-1 and HSF-1 are regulated in a similar manner (Figure 4F, quarter 1 and 3). Nevertheless, this comparative gene expression pattern does not reveal how they behave when all TFs sequester in the nucleus, i.e., under the rIIS condition. Therefore, further expression was considered in the rIIS condition (Figure 5A). Genes commonly regulated by DAF-16 with SKN-1 or HSF-1 under the rIIS condition (Figure 5A, serial no. 1–8) revealed that other TF regulatory capacity is undermined in the presence of DAF-16. DAF-16-independent genes regulated by SKN-1 and HSF-1 (Figure 4F) were expected to behave in a similar manner under the rIIS condition. Surprisingly, 57% and 20% of their shared target genes get repressed and activated differently under the rIIS condition (Figure 5A, serial no. 9–12). As the expression of these genes was not DAF-16-dependent, it hints at the involvement of some other factor under the rIIS condition whose actions may be strong enough to determine the direction of these genes’ expressions.

Next, we considered the commonly regulated genes by all three TFs under the rIIS condition (Figure A6D). Further, the grouping and association of these genes (N-226) with all possible combinations of TFs indicate that the direction of expression of these genes under the rIIS condition is determined by DAF-16, irrespective of the contribution made by SKN-1 and HSF-1 TFs (Figure 5B). This means that even the combined strength of SKN-1 and HSF-1 is not sufficient to change the direction of expression of these genes if DAF-16 sets to command them (Figure 5B, serial no. 4 and 5). This hints at the fact that it is the DAF-16 that controls the expression of common genes, and no other TF plays a significant role if the direction of expression is determined by DAF-16. Alternatively, it also implies that under the rIIS condition, if genes are majorly governed by SKN-1 or HSF-1, then DAF-16 may cooperate with them analogously; but for the genes that behave opposite to the nature of these TFs, DAF-16 leads and determines the direction of most of these genes. Together, our data suggest a sophisticated mechanism for balancing the expression of rIIS-dependent genes through these TFs (Figure 5C).

### 2.6. Molecular and Gene Regulatory Signatures of the TFs Targets Downstream of rIIS

**Figure 6:**
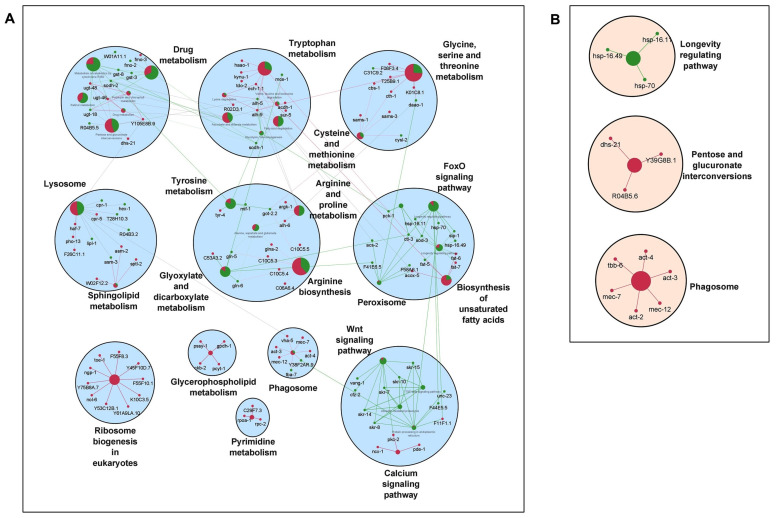
Gene networks of DAF-16 and SKN-1-regulated genes under rIIS conditions. (**A**) DAF-16-dependent pathway network. Most of the genes are activated in FoxO and calcium signaling, while ribosome biogenesis, pyrimidine metabolism, phagosome, and glycerophospholipids-related genes were found to be in a repressed condition. (**B**) SKN-1-dependent pathway network. Longevity regulating genes are activated while phagosome, pentose, and glucuronate interconversion genes remain repressed. Activated and repressed genes are shown by green and red colors, respectively.

**Figure 7:**
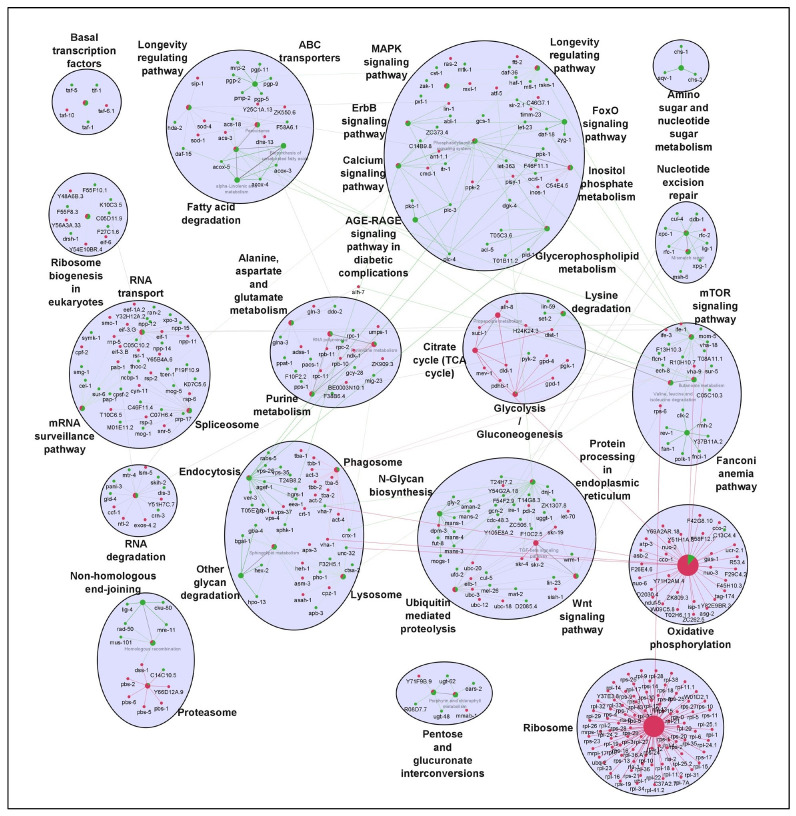
Gene network of HSF-1-regulated genes downstream of the rIIS condition. HSF-1 regulates multiple diverse pathways. Several pathways, including longevity, amino sugar, nucleotide sugar, ABC transporters, non-homologous end-joining, and mTOR signaling, mainly involve gene activation. Meanwhile, ribosome, proteosome, glycolysis, TCA cycle, and oxidative phosphorylation-related genes were found to be repressed by HSF-1. Activated and repressed genes are shown by green and red colors, respectively.

It is well established that longevity and its associated characteristics are multifactorial and involve complex interactions of different signaling mechanisms. To explore how the various molecular signatures involved are guided by these TFs, we built regulatory networks of genes regulated by rIIS downstream TFs (Figure 2B). To visualize the molecular interplay of each TF on different biological processes, individual TF-specific networks based on their target genes differential expression were created. We selected only significantly enriched clusters regulated by individual TFs (Figure 6 and Figure 7).

**Figure A7:**
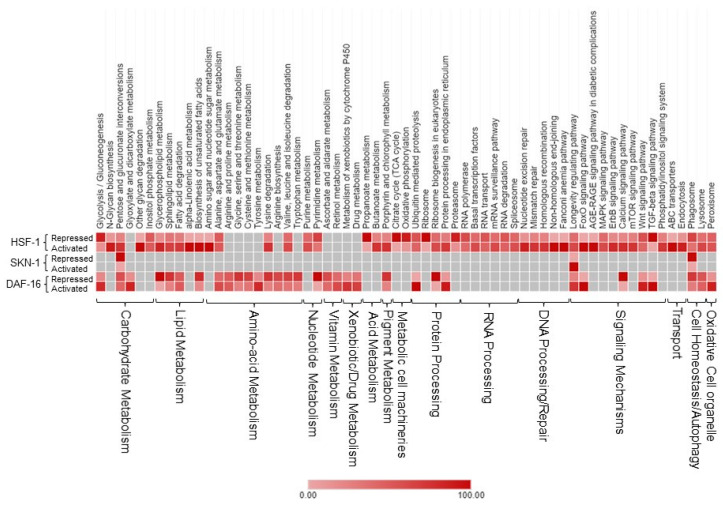
The relative percentage of differentially expressed genes regulated by the individual transcription factors are shown for each of the biological processes which were identified by gene network analysis.

For ease of understanding, these clusters were again re-grouped under more general terms, such as carbohydrate metabolism, lipid metabolism, amino-acid metabolism, RNA processing, etc. (Figure A7). We observed the maximum pathways/gene clusters to be regulated by HSF-1, followed by DAF-16, and a few by SKN-1 (Figure 6 and Figure 7). As expected, the genes involved in longevity were found to be regulated by all three TFs with maximum contribution by DAF-16 followed by SKN-1 and HSF-1 (Figure A7), as reported earlier [3,4,15]. Similarly, the role of these TFs in lifespan remodeling through autophagy and carbohydrate metabolism is well evident, also reflected in our study (Figure 6 and Figure 7). Genes controlling carbohydrate and lipid metabolic pathways were found to be dependent on DAF-16 and HSF-1. However, most of the amino-acid metabolism-regulating genes were DAF-16 dependent, except the branched-chain amino acid (valine, leucine, and isoleucine), and the conserved regulator of physiological aging was found to be primarily activated by both DAF-16 and HSF-1, as reported earlier [50]. DAF-16 activation is known to slow down the turnover of most proteins, which points towards the decreased abundance of the translational machinery [51]. Our data suggest that the genes required for translational machinery are also repressed at the transcription level by DAF-16 (Figure 6A). Moreover, many of these ribosomal genes were also found to be repressed by HSF-1 under rIIS (Figure 7).

To further evaluate these networks, a comparison among the proportion of gene associations per cluster was made where both contrasting and similar patterns of gene expression were observed for the regulation of different biological processes. We found that these TFs regulate many similar, as well as distinct, pathways. For instance, pentose and glucuronate interconversion and phagosomes are regulated by all TFs. Carbohydrate, lipid, and amino-acid metabolism, etc., are regulated both by DAF-16 and HSF-1. Specifically, glyoxylate and dicarboxylate metabolism, arginine, proline, glycine, serine, threonine, vitamins, and xenobiotic/drug metabolism are regulated primarily by DAF-16, while N-glycan, inositol phosphate, alpha-linolic acid, an amino sugar, purine, propanoate, RNA/DNA processing, etc., are primarily regulated by HSF-1. Moreover, similar pathways are also found to be regulated by opposingly different TFs. For example, glycolysis, glycerophospholipid, sphingolipid, biosynthesis of unsaturated fatty acids, lysine degradation, Wnt, and TGF-beta signaling pathways are regulated both by DAF-16 and HSF-1, but inversely. It could be due to their nonoverlapping set of genes involved in similar processes but regulated differently by distinct TFs. Thus, it shows the existence of a significant degree of overlap among regulatory functions despite their independent nature of gene regulation. Further, close observations in the networks indicate that the same genes differently controlled by these TFs are involved in regulating a particular biological process. For example, common genes *asm-3* and *sip-1* were found to be activated by DAF-16 and repressed by HSF-1 to regulate sphingolipid and longevity pathways, respectively. Conversely, some genes such as *rpc-2* and *sur-5* found to be repressed by DAF-16 and activated by HSF-1 were involved in the regulation of nucleotide and amino-acid (valine, leucine, and isoleucine) metabolism, respectively. In a nutshell, our study reveals several unidentified, as well as earlier reported, biological processes governed by the mechanisms regulating the complex biology of aging.

## 3. Discussion

There are several phenotypes associated with long-lived mutants, in addition to longevity, such as larval arrest, oxidative and heat stress resistance, pathogen resistance, reproduction, adult behavior, and metabolism [52,53]. In fact, the genes linked with these associated phenotypes were characterized first and subsequently found to be contributing to longevity modulation. The IIS pathway contains many evolutionarily conserved components, including downstream transcription factors that regulate most of these associated phenotypes in addition to prolonged longevity, but in a condition-specific manner [3,4,7]. Despite our extensive knowledge of the IIS pathway that leads to the activation of DAF-16, SKN-1, and HSF-1, the way TFs relay their transcriptional output in a comparable genetic condition to benefit the organism has long remained elusive. This study provides the first systematic rIIS-dependent TF target identification and regulation in a comparable experimental condition with the same genetic background.

Our data indicate that DAF-16 regulates a relatively small fraction of *C. elegans* genes, but under the rIIS condition, it regulates the majority. On the contrary, SKN-1 controls a large proportion of genes under normal conditions but a relatively smaller proportion under the rIIS condition. From the perspective of the total transcriptional response perspective, HSF-1 regulated genes were comparable to those of SKN-1, but under the rIIS condition, it regulates more than double the genes regulated by SKN-1. This implies that SKN-1 may have more important roles under normal conditions than in the rIIS condition, while the opposite is true for DAF-16. HSF-1, on the other hand, seems to play an important role both under normal and rIIS conditions. This seems plausible, as removing DAF-16 slightly affects many phenotypes, including lifespan in wild-type worms [54]. However, SKN-1 and HSF-1 removal affect many phenotypes, including development, normal lifespan, oxidative stress, pathogen resistance, and heat stress to a greater extent [55,56,57]. We presume this could be due to the different levels of TF molecules present in the nucleus under varied conditions. However, under basal conditions, the nucleus to cytoplasmic ratios of all three transcription factors was reported to be significantly lower as compared to the rIIS condition, where it increases mani-fold in the nucleus [2,3,7,58]. This hints that it is not only the dosage of the TFs translocated into the nucleus but may possibly be the active TF molecules that regulate the target gene expression in a context-specific manner. Therefore, it is possible that under the rIIS condition, a relatively smaller number of SKN-1 molecules translocated and/or are activated into the nucleus compared to DAF-16 and/or HSF-1, which corresponds to the lower number of genes regulated by them, as observed in this study. Moreover, the fraction of rIIS genes controlled by these TFs are suggestive of the dominant DAF-16 activity due to its specificity to the rIIS condition, but the primary role of SKN-1 seems to be the basal condition, while HSF-1 seems to be necessary for both scenarios.

Analysis of all possible dimensions of the DAF-16 regulated rIIS-dependent common genes suggests that the direction of such genes is entirely coupled with DAF-16. It reflected the invincible and dominant role of DAF-16 over other TFs under rIIS. Surprisingly, the DAF-16 was independent, but SKN-1- and HSF-1-regulated common genes, where both TFs individually regulate them in a similar manner (Figure 4F), were found to behave differently under rIIS conditions. It hints at the involvement of some unidentified factor other than DAF-16, which acts antagonistically to the SKN-1 and HSF-1 under the rIIS condition.

The understanding of the specificity and complexity of the target genes provides insight into how they act and are targeted by the transcription factors during lowered insulin signaling. This systematic resolution of the TF-dependent transcriptional network identifies common and specific molecular signatures, which may contribute to different phenotypes of the rIIS condition. Our study identified overlapping but distinct molecular niches by comprehensive and well-interconnected TF-associated regulatory networks. We were able to resolve the rIIS-dependent TFs transcriptional complexity, which itself provides a significant resource for future studies. Finally, we would like to note that all these TFs are evolutionarily well conserved. Hence, their regulatory roles, including their complexity, specificity, overlapping, as well as distinct molecular niche described here, may be conserved in other organisms, and their further exploration may eventually benefit our understanding of human aging and age-linked diseases.

## 4. Materials and Methods

### 4.1. Strain Maintenance

Strains of *rrf-3(pk1426)*/[RRID:WB-STRAIN:WBStrain00028995], *rrf-3(pk1426);daf-2(e1370)*/RRID:WB-STRAIN:WBStrain00004874, and *daf-2(e1368)*/RRID:WB-STRAIN:WBStrain00006381 mutant worms were obtained from the Caenorhabditis Genetics Centre (Minneapolis, MN, USA). Double mutants *rrf-3(pk1426);daf-2(e1368)* were made by crossing *rrf-3(pk1426)* males and *daf-2(e1368)* hermaphrodites using standard genetic techniques. Throughout the manuscript, *rrf-3(pk1426)* and *rrf-3(pk1426);daf-2(e1368)* on empty vector [*E. Coli* HT115 (DE3) carrying L4440 vector]/RRID:WB-STRAIN:WBStrain00041074 are referred to as “control” and “reduced insulin signaling (rIIS) condition”, respectively. Worms were grown at 20 °C unless otherwise mentioned. Animals were passaged frequently to avoid starvation and overcrowding during routine maintenance.

### 4.2. RNAi Plates Preparation

First, nematode growth medium (NGM) was prepared by mixing 3 g of NaCl (Merck Life Sciences Pvt. Ltd., Mumbai, India, Catalog #1.93606.0521), 2.5 g peptone (Himedia Laboratories, Mumbai, India, Catalog #RM001), and 17 g agar (Sisco Research Laboratories Pvt. Ltd., Mumbai, India, Catalog #24970) in 1 L double distilled water. After autoclaving and cooling down at 55–60 °C, 0.5 mL of cholesterol (Himedia Laboratories, Mumbai, India, Catalog #TC1101) [10 mg/mL in ethanol (Merck Life Sciences Pvt. Ltd., Mumbai, India, Catalog #1.00983.0511)], 1 mL of 1M CaCl_2_ (Thermo Fisher Scientific, Waltham, Massachusetts, United States, Catalog #C614-500), 1 mL of 1M MgSO_4_ (Himedia Laboratories, Mumbai, India, Catalog #GRM684), and 25 mL of 1M (pH 6.0) KPO_4_. KPO4 (1 M) was made by mixing 10.83 g of KH_2_PO_4_ (Sisco Research Laboratories Pvt. Ltd., Mumbai, India, Catalog #54358) and 3.56 g of K_2_HPO_4_ (Himedia Laboratories, Mumbai, India, Catalog #GRM1045) in 100 mL of double-distilled water. Then, NGM was supplemented with 100 µg/mL ampicillin (Bio Basic Inc., Markham, Ontario, Canada, Catalog #AB0028) and 2 mM IPTG (BR Biochem Life sciences Pvt. Ltd., New Delhi, India, Catalog #BC0168). After pouring, plates were dried at room temperature for 2–3 days. *E. coli* bacterial strain HT115 containing the gene of interest for RNAi in the L4440 vector construct was cultured in Luria Bertani (LB) media (Himedia Laboratories, Mumbai, India, Catalog #M1245) at 37 °C overnight in a shaker incubator. It was supplemented with 12.5 µg/mL tetracycline (Bio Basic Inc., Markham, Ontario, Canada, Catalog #TB0504) and 100 µg/mL ampicillin. The next day, overnight grown primary culture was inoculated in fresh LB media containing 100 µg/mL ampicillin in the ratio of 1:100 for secondary culture at 37 °C shaken until OD_600_ in the spectrophotometer (Shimadzu Corporation, Kyoto, Japan, Model #UV-1900) reached between 0.6–0.8. The secondary cultured bacterial cells were pelleted down by centrifuging at 5000× *g* for 10 min at 4 °C and resuspended in 1/10th volume of M9 buffer containing 1mM IPTG and100 µg/mL ampicillin. IPTG in the plates and M9 suspended culture was used to induce the T7 polymerase expression in the HT115 bacteria that transcribes the dsRNA in the plasmid. M9 culture suspension of 120 µL was seeded onto 60 mm RNAi plates and dried to develop a bacterial lawn at room temperature for nearly 2 days.

### 4.3. Synchronization of Worms by Hypochlorite Treatment

*C. elegans* worms were grown on *E. coli* OP50 bacteria (Caenorhabditis Genetics Centre, Minneapolis, MN, USA) until the egg-containing gravid adult stage. Worms from the plates were collected using M9 buffer in a 15 mL centrifuge tube. M9 buffer was made by mixing 6 g of Na_2_HPO_4_ (Himedia Laboratories, Mumbai, India, Catalog # TC051), 3 g of KH_2_PO_4_, 5 g of NaCl, and 0.25 g of MgSO_4_ in 1 L double distilled water. Worms were centrifuged in a swing bucket rotor (Eppendorf India Pvt. Ltd., Chennai, India, Model #5810R) at 1200× *g* for 60 s followed by resuspension of the worm pellet in M9 buffer. This washing procedure was repeated three times. Then, worm pellet was dissolved in a bleach solution [double distilled H_2_O, sodium hypochlorite (Merck Life Sciences Pvt. Ltd., Mumbai, India, Catalog #1.00983.0511), 5 N NaOH (Merck Life Sciences Pvt. Ltd., Mumbai, India, Catalog #1.06462.1000) in the ratio of 7:2:1]. To obtain hypochlorite-resistant eggs by dissolving gravid worm bodies, the above suspension was vortexed for 6–8 min in a vortex shaker (Tarsons, Kolkata, India, Model #Spinix). The eggs were washed 5–6 times by centrifuging at 2000 × g, then decanting the 1 × M9 with a suction pump (Rocker Scientific Co., Kaohsiung, Taiwan, Model #Rocker410) followed by resuspension in 1 × M9 buffer to remove traces of bleach and alkali. After the final wash, eggs were resuspended in 10 mL of M9 buffer and kept on a rocking shaker (Tarsons, Kolkata, India, Model #Rockymax) for 18–20 h at 21–22 °C for L1 offspring hatching and arrest. Hatching percentage was scored to confirm L1 synchronized animals’ viability. L1 arrested worms were seeded on RNAi plates to grow until the L4 stage. Unhealthy and un-synchronized animals, if any were discarded at L4 stage. Remaining healthy and synchronized animals were grown further until young adult/early gravid stage for RNA isolation.

### 4.4. Lifespan Assays of TFs Gene Inactivation by RNAi

Gravid adult worms grown on *E. coli* OP50 were bleached as described above. The eggs were kept on different RNAi plates to hatch. On reaching adulthood, 80–90 young adult worms were transferred in triplicates to the corresponding RNAi plates containing Fluorodeoxyuridine (FudR) (Sisco Research Laboratories Pvt. Ltd., Mumbai, India, Catalog #81015) to a final concentration of 0.1 mg/mL of agar. On the 7th day of adulthood, sick, undeveloped, sluggish, and slow-dwelling worms were removed from the life span population, and the remaining were considered for scoring. The age-synchronized population of worms was scored every alternate day until they died. They were considered dead when they failed to respond to external stimuli. Percentage survival was plotted against the number of days.

### 4.5. Worm Sample Prep for RNA Seq

L1 synchronized worms were grown in three biological replicates on empty vector and test RNAi plates. Worms were collected at YA/gravid stage for RNA isolation in a 15 mL centrifuge tube using 1 × M9 buffer and washed at least four times to remove bacterial contamination. Before final wash, the suspension was transferred to the 2 mL centrifuge tube, and 300 μL of Trizol (Invitrogen) reagent (ThermoFisher Scientific, Waltham, Massachusetts, United States, Catalog #15596026) was added to nearly 50–60 μL of the worm pellet. Samples were stored at −80 °C (Eppendorf North America, Connecticut, United States, Model #CryoCubeF740) until further processing. A few unhealthy, immotile animals in *rrf-3* strain were observed following heat shock, but we quickly removed most of them manually. After the RNA quality check, two biological replicates with the highest RIN value using automated electrophoresis (Agilent Technologies, Santa Clara, CA, United States, Model #Bioanalyzer2100) were selected for further downstream processing.

### 4.6. RNA Isolation

Frozen worms at −80 °C were lysed with three freeze-thaw cycles and intermittent vortexing in liquid nitrogen. To isolate RNA, 150 μL of chloroform (Merck Life Sciences Pvt. Ltd., Mumbai, India, Catalog #1.07024.0521) was added to the worm pellets, and tubes were gently inverted several times. After incubation for 3 min at room temperature, samples were centrifuged at 12,000× *g* for 15 min at 4 °C. The upper aqueous phase was gently removed into a fresh tube. An equal volume of isopropanol (Fisher Scientific, Ottawa, ON, Canada, Catalog #BP2618-500) was added, and the reaction was allowed to sit at room temperature for 10 min. After centrifugation at 12,000× *g* for 10 min at 4 °C, supernatants were carefully discarded, and the remaining pellets were washed using 1 mL 70% ethanol. After centrifugation at 12,000× *g* for 5 min at 4 °C, RNA pellets were dried at room temperature and then dissolved in nuclease-free water. It was kept at 65 °C for 10 min with intermittent tapping. RNA concentration was determined by fluorimeter (Invitrogen, California, United States, Model #Qubit3.0), and quality was checked using Bioanalyzer with RNA kit (Agilent Technologies, United States, Catalog #RNA6000Nano).

### 4.7. Real-Time Quantitative Reverse Transcription PCR (qRT-PCR)

RNAi knockdown efficiency of TF genes and validation of NGS data by selecting well-known genes was completed by quantifying their expression levels with Real-Time quantitative Reverse Transcription PCR (qRT-PCR). Complementary DNA (cDNA) was prepared using SuperScript III First-Strand Synthesis System Kit (ThermoFisher Scientific, Waltham, MA, United States, Catalog #18080051). Briefly, 1 µg of total RNA was used for cDNA preparation. Oligo dT primers and dNTPs (supplied with cDNA preparation kit) were mixed with it. The mixed solution was heated at 65 °C for 5 min followed by cooling at 4 °C for 1 min. To this mixture, dithiothreitol RNase OUT, 5 × reverse transcriptase buffer, and 1 μL/reaction of Superscript Reverse transcriptase III enzymes (supplied with cDNA preparation kit) were added in the required concentrations. The reaction was incubated at 42 °C for 50 min and later terminated by incubation at 70 °C for 15 min. Quantification of genes was done by qRT-PCR using Brilliant III Ultra-Fast SYBR QPCR Master Mix (Agilent Technologies, California, United States, Catalog #600882) and Real-Time PCR system (Bio-Rad, California, United States, Model #CFX96 Touch Real-Time PCR Detection System) as per the manufacturer’s guidelines. The relative expression of genes was calculated according to the ΔΔCt method [59], where ΔCts of genes were obtained after normalization with Ct of actin. Primers used for quantitative RT-PCR are listed in Table S3.

### 4.8. RNA Sequencing and Analysis

Two biological replicates with RIN (RNA integrity number) values above 9 were selected for the study. The cDNA libraries were constructed with TruSeq RNA Library Prep Kit v2 (Illumina Inc., California, United States). Sequencing (72 or 50 bp single end) was performed using NGS platforms (Illumina Inc. California, United States, Models #Genome Analyzer IIx or HiSeq 2500 systems). Imaging, base calling, and quality scoring were done as per standard manufacturer’s guidelines (Illumina Inc.). The demultiplexing and conversion of BCL file format reads to FASTQ file format was done with the Illumina-supported CASAVA v1.8.2 software package. Read counts were then aligned to the reference genome (WBcel 235), and their normalized abundances were calculated as Reads Per Kilobase Million (RPKM) using graphic user interface NGS data analysis package (Qiagen, Germantown, Maryland, United States, Tool version # CLC Genomics Workbench v12.0.3). Gene fold changes were calculated among samples based on negative binomial Generalized Linear Models (GLM), which corrects for differences in library size between samples and the effects of confounding factors. Genes with an absolute fold change of at least 1.5 and standard *p*-values below 0.05 were considered as differentially expressed. To evaluate variance among biological replicates and conditions, normalized log counts per million (CPM) values and z-score normalization across samples for each gene were applied followed by PCA analysis and hierarchical clustering of the Euclidean distances [60].

### 4.9. Transcriptional Regulatory Network Analysis

Distinct TF-specific transcriptional regulatory networks were built by considering differentially expressed genes using Cytoscape v3.8.2 [61]. To visualize the non-redundant biological terms and for biological interpretation of a large set of genes in a functionally grouped network, Cytoscape plug-in ClueGO v2.5.7 was used [62]. Genes for TF-specific networks were made by selecting parameters of style as clusters, ontologies pathways-KEGG, a *p*-value of pathways ≤0.05, and by keeping other parameters at default values. Activated and repressed genes were represented with green and red color nodes and edges, respectively. Identification of individual clusters and annotation with an enclosing shape and labels were completed with the semi-automated Cytoscape plug-in AutoAnnotate v1.3.4 [63]. Individual clusters were further separated and aligned manually for clear representation.

### 4.10. Statistical Analysis

Basic survival analysis with the statistical test was conducted using the Mantel-Cox test using Oasis software available at (http://sbi.postech.ac.kr/oasis; Accessed on 9 May 2018). The difference in survival with a *p*-value ≤ 0.05 was considered significant. Differential expressed genes by RNA-Seq data were determined with standard *p*-values calculated by Baggerley et al.’s test [64]. Genes with a *p*-value ≤ 0.05 were considered with significant expression change. In all the Venn diagrams, hypergeometric *p*-values were calculated by online software (http://www.geneprof.org/GeneProf/tools/hypergeometric.jsp; accessed on 17 April 2020), and their exact *p*-values are indicated in the text as well as in figures. Statistical analysis of quantitative real-time PCR was performed using online statistical software (Systat Software Inc., Chicago, IL, United States, Tool version #SigmaPlot10.0; Accessed on 06-08-2021) with an unpaired two-tailed Student’s *t*-test. These values were represented with * ≤0.05, ** ≤0.01 and *** ≤0.001.

## Data Availability

Raw RNA-Seq data have been deposited in the Gene Expression Omnibus (GEO)-NCBI-NIH database under the Super Series accession number GSE184415. The list of genes generated for detailed analysis shown in the manuscript is being provided as processed data files.

## Supplementary Materials

The following are available online at https://www.mdpi.com/article/10.3390/ijms222212462/s1.
